# Racial/Ethnic Disparities in Very Preterm Birth and Preterm Birth Before and During the COVID-19 Pandemic

**DOI:** 10.1001/jamanetworkopen.2021.1816

**Published:** 2021-03-17

**Authors:** Teresa Janevic, Kimberly B. Glazer, Luciana Vieira, Ellerie Weber, Joanne Stone, Toni Stern, Angela Bianco, Brian Wagner, Siobhan M. Dolan, Elizabeth A. Howell

**Affiliations:** 1 Blavatnik Family Women’s Health Research Institute, Icahn School of Medicine at Mount Sinai, New York, New York; 2 Department of Obstetrics, Gynecology and Reproductive Science, Icahn School of Medicine at Mount Sinai, New York, New York; 3 Department of Population Health Science and Policy, Icahn School of Medicine at Mount Sinai, New York, New York; 4 Department of Obstetrics and Gynecology, Perelman School of Medicine, University of Pennsylvania, Philadelphia

## Abstract

**Importance:**

The coronavirus disease 2019 (COVID-19) pandemic may exacerbate existing racial/ethnic inequities in preterm birth.

**Objective:**

To assess whether racial/ethnic disparities in very preterm birth (VPTB) and preterm birth (PTB) increased during the first wave of the COVID-19 pandemic in New York City.

**Design, Setting, and Participants:**

This cross-sectional study included 8026 Black, Latina, and White women who gave birth during the study period. A difference-in-differences (DID) analysis of Black vs White disparities in VPTB or PTB in a pandemic cohort was compared with a prepandemic cohort by using electronic medical records obtained from 2 hospitals in New York City.

**Exposures:**

Women who delivered from March 28 to July 31, 2020, were considered the pandemic cohort, and women who delivered from March 28 to July 31, 2019, were considered the prepandemic cohort. Reverse transcription–polymerase chain reaction tests for the presence of severe acute respiratory syndrome coronavirus 2 (SARS-CoV-2) were performed using samples obtained via nasopharyngeal swab at the time of admission.

**Main Outcomes and Measures:**

Clinical estimates of gestational age were used to calculate VPTB (<32 weeks) and PTB (<37 weeks). Log binomial regression was performed to estimate Black vs White risk differences, pandemic cohort vs prepandemic cohort risk difference, and an interaction term representing the DID estimator. Covariate-adjusted models included age, insurance, prepregnancy body mass index, and parity.

**Results:**

Of 3834 women in the pandemic cohort, 492 (12.8%) self-identified as Black, 678 (17.7%) as Latina, 2012 (52.5%) as White, 408 (10.6%) as Asian, and 244 (6.4%) as other or unspecified race/ethnicity, with approximately half the women 25 to 34 years of age. The prepandemic cohort comprised 4192 women with similar sociodemographic characteristics. In the prepandemic cohort, VPTB risk was 4.4% (20 of 451) and PTB risk was 14.4% (65 of 451) among Black infants compared with 0.8% (17 of 2188) VPTB risk and 7.1% (156 of 2188) PTB risk among White infants. In the pandemic cohort, VPTB risk was 4.3% (21 of 491) and PTB risk was 13.2% (65 of 491) among Black infants compared with 0.5% (10 of 1994) VPTB risk and 7.0% (240 of 1994) PTB risk among White infants. The DID estimators indicated that no increase in Black vs White disparities were found (DID estimator for VPTB, 0.1 additional cases per 100 [95% CI, −2.5 to 2.8]; DID estimator for PTB, 1.1 fewer case per 100 [95% CI, −5.8 to 3.6]). The results were comparable in covariate-adjusted models when limiting the population to women who tested negative for SARS-CoV-2. No change was detected in Latina vs White PTB disparities during the pandemic.

**Conclusions and Relevance:**

In this cross-sectional study of women who gave birth in New York City during the COVID-19 pandemic, no evidence was found for increased racial/ethnic disparities in PTB, among women who tested positive or tested negative for SARS-CoV-2.

## Key Points

**Question:**

Was the first wave of the coronavirus disease 2019 (COVID-19) pandemic associated with exacerbated racial/ethnic disparities in preterm birth in New York City?

**Findings:**

This cross-sectional study found that racial/ethnic disparities in very preterm birth and preterm birth among 8026 women were similar during the first wave of the COVID-19 pandemic in New York City compared with the same period the year prior.

**Meaning:**

Monitoring of racial/ethnic disparities in adverse birth outcomes as the COVID-19 pandemic continues is warranted.

## Teaser

This cross-sectional study uses a difference-in-differences analysis to assess whether racial/ethnic disparities in very preterm birth or preterm birth increased in the first wave of the coronavirus disease 2019 pandemic vs the year prior to the pandemic among women who delivered in New York City.

## Introduction

Black women and infants experience persistent disadvantage in birth outcomes in the US. Black infants are 50% more like to be born preterm and twice as likely to be born very preterm.^1^ In New York City (NYC), Latina women are also at increased risk of delivering preterm.^2^ The coronavirus disease 2019 (COVID-19) pandemic threatens to exacerbate existing preterm birth (PTB) and very preterm birth (VPTB) disparities, yet data are scarce to inform this pressing concern.

The COVID-19 pandemic is replicating existing structures of inequality and disproportionately harming communities of color. Black and Latina women are more likely to be infected with severe acute respiratory syndrome coronavirus 2 (SARS-CoV-2) at delivery^3^ and more likely to experience pandemic-related psychosocial and economic impacts during pregnancy.^4^ Research thus far on obstetric outcomes during the pandemic typically report a modest increased risk of PTB among women with COVID-19^5^ and little to no increased risk among women who are asymptomatic but test positive for SARS-CoV-2.^6^ To date, research has not adequately examined the association of the COVID-19 pandemic with PTB from a health equity perspective.

To fill this gap, we conducted a difference-in-differences (DID) analysis of electronic medical records of 8026 women from 2 hospitals in a NYC health system, which draws patients from the Bronx, Manhattan, Queens, and Brooklyn. We compared racial/ethnic differences in PTB during the first wave of the pandemic with the year prior.

## Methods

### Study Design

We created a pandemic cohort of 3834 women who delivered between March 28, 2020, the date universal testing of women undergoing labor and delivery began, and July 31, 2020. We did not include births prior to universal testing owing to unknown SARS-CoV-2 status. We created a prepandemic cohort of 4192 women who delivered children from March 28 to July 31, 2019 (eFigure in the Supplement). We followed the Strengthening the Reporting of Observational Studies in Epidemiology (STROBE) reporting guideline for cross-sectional studies. The institutional review board of the Icahn School of Medicine at Mount Sinai approved the study and waived the requirement for obtaining informed consent because risk to participants was considered minimal and the study could not reasonably been conducted otherwise. No one received compensation or was offered any incentive for participating in this study.

Reverse transcription polymerase chain reaction tests for the presence of SARS-CoV-2 were performed using samples obtained via nasopharyngeal swab. We used electronic medical records to ascertain all variables. Participant race and ethnicity were self-reported on admission and classified according to the US Office of Management and Budget standards. We estimated VPTB (<32 weeks completed gestation) and PTB (<37 weeks completed gestation) using the clinician’s best estimate of gestational age.

### Statistical Analysis

We used log binomial regression to estimate a DID equation with main effects for Black vs White risk difference, pandemic vs prepandemic cohort risk difference, and an interaction term representing the DID estimator. The DID estimator estimates the additional disparity resulting from the pandemic beyond disparities that had previously existed. We repeated the model for Latina vs White women, restricting the pandemic cohort to positive or negative SARS-CoV-2 test status, and singleton births. We estimated multivariable models adjusting DID estimates for age, insurance type, prepregnancy body mass index, and parity. The DID approach is typically robust to confounding given the balance of covariates between treatment groups is constant over time. In multivariable analyses, we excluded observations with missing values (<4% for body mass index, <3% for polymerase chain reaction, and <1% all others). All analyses were conducted using SAS, version 9.4 (SAS Institute Inc). A 2-sided *P* < .05 was considered statistically significant.

## Results

The pandemic cohort included 3834 women; 492 (12.8%) identified as Black, 678 (17.7%) as Latina, 2012 (52.5%) as White, 408 (10.6%) as Asian, and 244 (6.4%) as another or unspecified race/ethnicity (Table 1). Roughly half of women in the pandemic cohort were aged 25 to 34 years. The prepandemic cohort (n = 4192) was similar to the pandemic cohort in sociodemographic characteristics (Table 1; *P* > .05 for all χ^2^ tests). Study characteristics also did not change over time within racial/ethnic groups (Table 2). Of 3731 women in the pandemic cohort, 210 (5.6%) tested positive for SARS-CoV-2. The risk of PTB was 14.4% (65 of 451) among Black births and 7.1% (156 of 2188) among White births during the prepandemic period, and 13.2% (65 of 491) among Black births and 7.0% (140 of 1994) among White births in the pandemic cohort (Table 3). There was no change in the Black vs White PTB disparity associated with the COVID-19 pandemic (DID estimator, 1.1 fewer cases per 100 [95% CI, −5.8 to 3.6]). The risk of VPTB was 4.4% (20 of 451) among Black births and 0.8% (17 of 2188) among White births in the prepandemic period, and 4.3% (21 of 491) among Black births and 0.5% (10 of 1994) among White births in the pandemic period. The DID estimator was 0.1 (95% CI, −2.5 to 2.8), indicating no change in the Black vs White VPTB disparity. We also did not find increases in Latina vs White PTB or VPTB disparities (Table 3). Covariate-adjusted estimates were similar (Table 3). Analyses stratified by SARS-CoV-2 status found DID estimators in the SARS-CoV-2–negative group were similar to the overall cohort (Table 4). We did not conduct DID analyses in the SARS-CoV-2–positive group owing to low counts of outcomes (Table 5). The DID estimators for VPTB and PTB were similar for singleton births.

**Table 1.** Sample Sociodemographic and Clinical Characteristics by COVID-19 Pandemic Cohort and SARS-CoV-2 Status

Characteristic	No. (%) of participants			
	Cohort before pandemic (March 28 to July 31, 2019)	Cohort during pandemic (March 28 to July 31, 2020)	Pandemic cohort with PCR test (n = 3731)^a^	
			Positive for SARS-CoV-2	Negative for SARS-CoV-2
No.	4192	3834^b^	210	3508
Age, y				
<25	416 (9.9)	458 (12.0)	32 (15.2)	402 (11.5)
25-34	2208 (52.7)	1953 (50.9)	104 (49.5)	1800 (51.3)
≥35	1567 (37.4)	1423 (37.1)	74 (35.2)	1306 (37.2)
Race/ethnicity				
Non-Latina Black	452 (10.8)	492 (12.8)	32 (15.2)	446 (12.7)
Latina	690 (16.5)	678 (17.7)	57 (27.1)	603 (17.2)
Non-Latina				
Asian	404 (9.6)	408 (10.6)	6 (2.9)	393 (11.2)
White	2205 (52.6)	2012 (52.5)	102 (48.6)	1842 (52.5)
Other or missing^c^	440 (10.5)	244 (6.4)	13 (6.2)	224 (6.4)
Insurance				
Private	3196 (76.2)	2902 (75.7)	144 (68.6)	2674 (76.2)
Medicaid	675 (16.1)	664 (17.3)	52 (24.8)	588 (16.8)
Medicare	50 (1.2)	28 (0.73)	1 (0.48)	26 (0.74)
Other	181 (4.3)	164 (4.3)	8 (3.8)	152 (4.3)
Self-pay	90 (2.2)	76 (2.0)	5 (3.1)	68 (1.9)
Multiple gestation	176 (4.2)	164 (4.3)	10 (4.8)	146 (4.2)
Parity				
Nulliparous	2045 (48.8)	1864 (48.6)	75 (35.7)	1766 (50.3)
Multiparous	2147 (51.2)	1970 (51.4)	135 (64.3)	1742 (49.7)
BMI				
Underweight (<18.5)	91 (2.2)	75 (2.0)	3 (1.4)	70 (2.0)
Normal weight (≤18.5 to <25)	1611 (38.4)	1419 (37.0)	61 (29.1)	1326 (37.8)
Overweight (25 to <30)	1313 (31.3)	1228 (32.0)	75 (35.7)	1114 (31.8)
Class 1 or 2 obesity (30 to <40)	884 (21.1)	819 (21.4)	54 (25.7)	724 (20.6)
Class 3 obesity (≥40)	136 (3.2)	118 (3.1)	7 (3.3)	110 (3.1)
Gestational age at delivery, wk				
Very preterm (<32)	62 (1.5)	53 (1.4)	0 (0)	53 (1.5)
Preterm (32-36)	303 (7.3)	277 (7.3)	21 (10.0)	246 (7.0)
Term				
Early (37-38)	1067 (25.6)	957 (25.1)	62 (29.5)	863 (24.6)
Full (39-40)	2411 (57.9)	2236 (58.8)	106 (50.5)	2064 (58.8)
Late (41)	318 (7.6)	277 (7.3)	19 (9.1)	250 (7.1)
Post (≥42)	6 (0.14)	6 (0.16)	0 (0)	6 (0.17)

Abbreviations: BMI, body mass index (calculated as weight in kilograms divided by height in meters squared); COVID-19, coronavirus disease 2019; PCR, polymerase chain reaction; SARS-CoV-2, severe acute respiratory syndrome coronavirus 2.

^a^ Sum of positive and negative cases is not equal to total number of births in cohort because 116 deliveries were missing SARS-CoV-2 PCR test data.

^b^ Column percentages do not sum to 100 in all cases owing to missing data.

^c^ Other race/ethnicity includes American Indian or Alaska Native, Native Hawaiian or Pacific Islander, and other or unspecified race/ethnicity.

**Table 2.** Sample Characteristics by Race/Ethnicity and Pandemic Cohort

Characteristic	Non-Latina Black (n = 944)			Latina (n = 1368)			Non-Latina White (n = 4217)		
	No. (%) of participants		*P* value	No. (%) of participants		*P* value	No. (%) of participants		*P* value
	Before pandemic	Pandemic		Before pandemic	Pandemic		Before pandemic	Pandemic	
No.	452	492		690	678		2205	2012	
Age, y									
<25	80 (17.7)	102 (20.7)	.34	113 (16.4)	129 (19.0)	.16	178 (8.1)	180 (9.0)	.54
25-34	244 (54.0)	244 (49.6)		397 (57.4)	356 (52.5)		1114 (50.5)	1021 (50.8)	
≥35	128 (28.3)	146 (29.7)		180 (26.1)	193 (28.5)		912 (41.4)	811 (40.3)	
Insurance									
Private	232 (51.3)	270 (54.9)	.81	343 (49.7)	333 (49.1)	.89	1953 (88.6)	1783 (88.6)	.10
Medicaid	175 (38.7)	178 (36.2)		292 (42.3)	298 (44.0)		103 (4.7)	104 (5.2)	
Medicare	10 (2.2)	8 (1.6)		4 (0.58)	4 (0.59)		28 (1.3)	10 (0.50)	
Other	14 (3.1)	16 (3.3)		23 (3.3)	22 (3.2)		93 (4.2)	91 (4.5)	
Self-pay	21 (4.7)	20 (4.1)		28 (4.1)	21 (3.1)		28 (1.3)	24 (1.2)	
Multiple gestation	31 (6.9)	26 (5.3)	.31	26 (3.8)	26 (3.8)	.95	101 (4.6)	90 (4.5)	.87
Parity									
Nulliparous	219 (48.5)	230 (46.8)	.60	294 (42.6)	296 (43.7)	.70	1052 (47.7)	945 (47.0)	.63
Multiparous	233 (51.6)	262 (53.3)		396 (57.4)	382 (56.3)		1153 (52.3)	1067 (53.0)	
Body mass index^a^									
Underweight (<18.5)	7 (1.6)	6 (1.2)	.75	6 (0.87)	8 (1.2)	.96	52 (2.4)	36 (1.8)	.18
Normal weight (18.5 to <25)	95 (21.0)	108 (22.0)		158 (22.9)	160 (23.6)		953 (43.2)	861 (42.8)	
Overweight (25 to <30)	135 (29.9)	146 (29.7)		219 (31.5)	219 (32.3)		723 (32.8)	668 (33.2)	
Class 1 or 2 obesity (30 to <40)	156 (34.5)	172 (35.0)		234 (33.9)	220 (32.5)		379 (17.2)	326 (16.2)	
Class 3 obesity (≥40)	43 (9.5)	24 (4.9)		41 (5.9)	36 (5.3)		32 (1.5)	36 (1.8)	

^a^ Calculated as weight in kilograms divided by height in meters squared.

**Table 3.** Difference-in-Differences Analysis of Black vs White and Latina vs White Disparities in Preterm and Very Preterm Births

Outcome	Before pandemic (March 28 to July 31, 2019)			Pandemic (March 28 to July 31, 2020)			Risk difference before vs during pandemic, % (95% CI)
	Denominator	Cases, No.	Risk, %	Denominator	Cases, No.	Risk, %	
**Black vs White births**							
Preterm birth							
Non-Latina White	2188	156	7.1	1994	140	7.0	−1.1 (−1.7 to 1.5)
Non-Latina Black	451	65	14.4	491	65	13.2	−1.2 (−5.6 to 3.2)
Difference			7.3			6.2	−1.1 (−5.8 to 3.6)
Adjusted difference^a^^,^^b^							−1.0 (−5.8 to 3.8)
Very preterm birth		5124					
Non-Latina White	2188	17	0.8	1994	10	0.5	−0.3 (−0.8 to 0.2)
Non-Latina Black	451	20	4.4	491	21	4.3	−0.2 (−2.8 to 2.5)
Difference			3.7			3.8	0.1 (−2.5 to 2.8)
Adjusted difference^a^^,^^b^							0.3 (−2.5 to 3.1)
**Latina vs White births**							
Preterm birth							
Non-Latina White	2188	156	7.1	1994	140	7.0	−0.1 (−1.7 to 1.5)
Latina	688	74	10.8	674	67	9.9	−0.8 (−4.1 to 2.4)
Difference			3.6			2.9	−0.7 (−4.3 to 2.9)
Adjusted difference^a^^,^^b^							−1.0 (−4.6 to 2.7)
Very preterm birth		5124					
Non-Latina White	2188	17	0.8	1994	10	0.5	−0.3 (−0.8 to 0.2)
Latina	688	10	1.5	674	9	1.3	−0.1 (−1.4 to 1.1)
Difference			0.7			0.8	0.2 (−1.2 to 1.5)
Adjusted difference^a^^,^^b^							0.2 (−1.3 to 1.6)

^a^ Adjusted for maternal age (continuous), parity (nulliparous, multiparous), prepregnancy body mass index (BMI; calculated as weight in kilograms divided by height in meters squared) (BMI <25, BMI ≥25), and insurance coverage (Medicaid, private, and other or self-pay); model for Black vs White very preterm birth disparity not adjusted for insurance coverage owing to nonconvergence.

^b^ Observations with missing covariate values dropped from adjusted analyses (<4% for BMI, <1% all others); 20 births in prepandemic cohort (1 Black, 2 Latina, and 17 White births) and 23 in pandemic cohort (1 Black, 4 Latina, and 18 White births) excluded because of missing gestational age.

**Table 4.** Difference-in-Differences Analysis of Racial/Ethnic Disparities in Preterm and Very Preterm Births Among Women Testing Negative for SARS-CoV-2

Outcome	Before pandemic (March 28 to July 31, 2019)			Pandemic (March 28 to July 31, 2020)			Risk difference before vs during pandemic, % (95% CI)
	Denominator	Cases, No.	Risk, %	Denominator	Cases, No.	Risk, %	
**Black vs White births**							
Preterm birth							
Non-Latina White	2188	156	7.1	1824	127	7.0	−0.2 (−1.8 to 1.4)
Non-Latina Black	451	65	14.4	445	59	13.3	−1.2 (−5.7 to 3.4)
Difference			7.3			6.3	−1.0 (−5.8 to 3.8)
Adjusted difference^a^^,^^b^							−0.9 (−5.8 to 4.0)
Very preterm birth		5124					
Non-Latina White	2188	17	0.8	1824	10	0.6	−0.2 (−0.7 to 0.3)
Non-Latina Black	451	20	4.4	445	21	4.7	0.3 (−2.5 to 3.0)
Difference			3.7			4.2	0.5 (−2.3 to 3.3)
Adjusted difference^a^^,^^b^							0.7 (−2.2 to 3.6)
**Latina vs White births**							
Preterm birth							
Non-Latina White	2188	156	7.1	1824	127	7.0	−0.2 (−1.8 to 1.4)
Latina	688	74	10.8	600	61	10.2	−0.6 (−3.9 to 2.8)
Difference			3.6			3.2	−0.4 (−4.1 to 3.3)
Adjusted difference^a^^,^^b^							−0.6 (−4.4 to 3.1)
Very preterm birth							
Non-Latina White	2188	17	0.8	1824	10	0.6	−0.2 (−0.7 to 0.3)
Latina	688	10	1.5	600	9	1.5	0.0 (−1.3 to 1.4)
Difference			0.7			0.9	0.3 (−1.1 to 1.7)
Adjusted difference^a^^,^^b^							0.3 (−1.2 to 1.7)

Abbreviation: SARS-CoV-2, severe acute respiratory syndrome coronavirus 2.

^a^ Adjusted for maternal age (continuous), parity (nulliparous, multiparous), prepregnancy body mass index (BMI; calculated as weight in kilograms divided by height in meters squared) (BMI <25, BMI ≥25), and insurance coverage (Medicaid, private, and other or self-pay); model for Black vs White very preterm birth disparity not adjusted for insurance coverage owing to nonconvergence.

^b^ Observations with missing covariate values dropped from adjusted analyses (<4% for BMI, <1% all others).

**Table 5.** Difference-in-Differences Analysis of Racial/Ethnic Disparities in Preterm and Very Preterm Birth Among Women Testing Positive for SARS-CoV-2^a^

Outcomes	Before pandemic (March 28 to July 31, 2019)			Pandemic (March 28 to July 31, 2020)		
	Denominator	Cases, No.	Risk, %	Denominator	Cases, No.	Risk, %
**Black vs White births**						
Preterm birth						
Non-Latina White	2188	156	7.1	102	11	10.8
Non-Latina Black	451	65	14.4	32	4	12.5
Difference			7.3			1.7
Very preterm birth						
Non-Latina White	2188	17	0.8	102	0	0.0
Non-Latina Black	451	20	4.4	32	0	0.0
Difference			3.6			0.0
**Latina vs White births**						
Preterm birth						
Non-Latina White	2188	156	7.1	102	11	10.8
Latina	688	74	10.8	56	3	5.4
Difference			3.6			−5.4
Very preterm birth		5124				
Non-Latina White	2188	17	0.8	102	0	0.0
Latina	688	10	1.5	56	0	0.0
Difference			0.6			0.0

^a^ Difference-in-differences regression analyses not conducted for severe acute respiratory syndrome coronavirus 2 (SARS-CoV-2)–positive groups owing to low outcome counts.

## Discussion

We found no evidence that the first wave of the COVID-19 pandemic increased racial/ethnic disparities in preterm birth in NYC. Results were similar by SARS-CoV-2 status.

Our findings should be considered in the context of a current hypothesis that the lockdown has lessened the risk of PTB for women.^7,8,9^ In contrast to this hypothesis, in Philadelphia, PTB did not change,^10^ whereas in California, VPTB increased slightly among Latina mothers.^11^ Unlike other recent reports,^10,11^ we explicitly tested racial/ethnic disparities with a robust DID design and were able to stratify our results by active SARS-CoV-2 infection.

Researchers have proposed potential reasons for a decrease in PTB during the COVID-19 pandemic, such as a decrease in known risk factors for PTB, including occupational environment, pollution, or stress.^9^ However, any benefit from COVID-19–related restrictions may be less prevalent among Black and Latina women in NYC, who may be more likely to be essential workers^12^ and to experience higher rates of COVID-19 pandemic–related stress, anxiety, and food insecurity.^4,13,14^ Black and Latina women are also more likely than White women to experience loss and trauma due to COVID-19.^15^ Decreased access to prenatal care, increased incidence of pregnancy complications, or decreased control of chronic conditions may also play a role. Despite these potential mechanisms, we did not find an increase in racial/ethnic differences in PTB. Regardless, given the known inequitable repercussions of COVID-19 in Black and Latinx populations, continued monitoring of racial/ethnic disparities in preterm birth is warranted.

### Limitations and Strengths

The limitations of our study include the lack of information on maternal comorbidities and SARS-CoV-2 infections prior to delivery. If healthy White women disproportionately left NYC to deliver their infants during the pandemic, this selection bias would cause a greater proportion of preterm births in the White pandemic cohort, and underestimate an increase in PTB disparities. Evaluation of covariates by race/ethnicity and cohort suggested this bias was minimal. Another limitation is the lack of precision to calculate DID estimators among women who tested positive for SARS-CoV-2. Our results may not be generalizable to nonurban settings. The strengths of our study include a diverse population with a rigorous DID analysis design.

## Conclusions

In this cross-sectional study of women who gave birth in NYC during the COVID-19 pandemic, we found no evidence of increased racial/ethnic differences in preterm birth, among women who tested positive or negative for SARS-CoV-2. However, continued monitoring of birth inequities as the pandemic continues is warranted.

## Notes

Supplement. **eFigure.** Study Population Flow Diagram
