# Supplementary material for: Racial/Ethnic Disparities in Very Preterm Birth and Preterm Birth Before and During the COVID-19 Pandemic
Source: JAMA Netw Open. 2021 Mar 17;4(3):e211816. doi: 10.1001/jamanetworkopen.2021.1816 (PMC7970336; doi:10.1001/jamanetworkopen.2021.1816)
Supplement: Supplement. — eFigure. Study Population Flow Diagram [file jamanetwopen-e211816-s001.pdf]

Supplementary Online Content

Janevic T, Glazer KB, Vieira L, et al. Racial/ethnic disparities in very preterm birth and preterm birth before and during the COVID-19 pandemic. *JAMA Netw Open*. 2021;4(3):e211816.
doi:10.1001/jamanetworkopen.2021.1816

eFigure. Study Population Flow Diagram

This supplementary material has been provided by the authors to give readers additional information about their work.

© 2021 Janevic T et al. *JAMA Network Open*.

eFigure. Study Population Flow Diagram

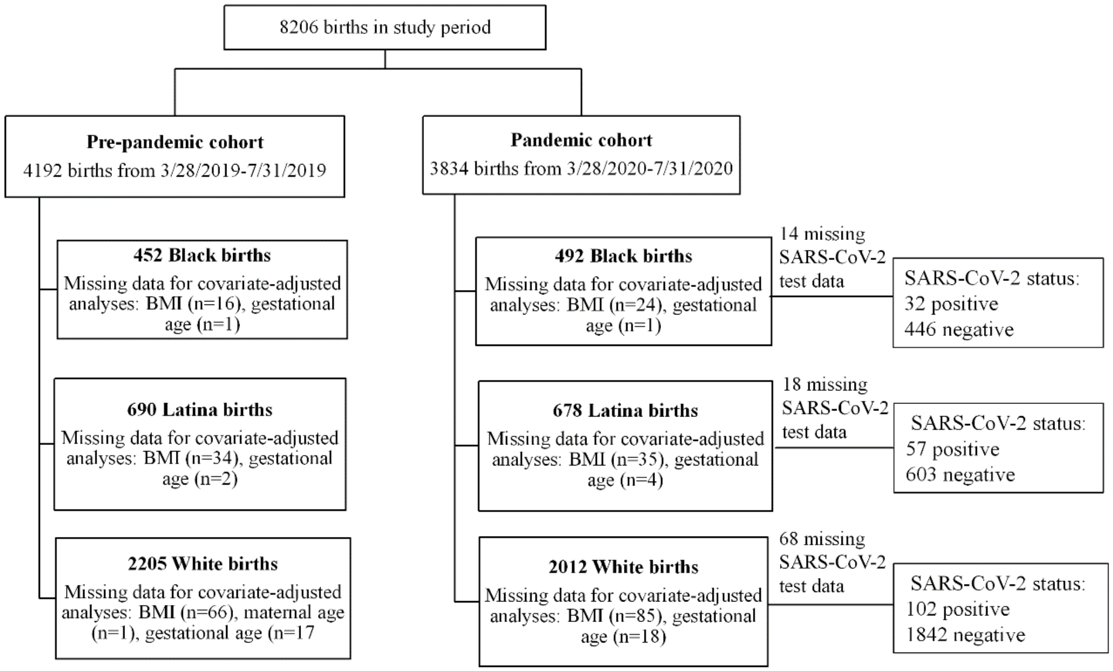

Study population drawn from electronic medical records of all deliveries in two hospitals in a NYC health system within the study period (3/28-7/31/2019, 3/28-7/31/2020), excluding births to women with protected status (HIV+ women, hospital employees, VIP).

© 2021 Janevic T et al. *JAMA Network Open*.
